# Specific Instruction in the Psychology and Hygiene of Sex

**Published:** 1913-02

**Authors:** 


### Specific Instruction in the Psychology and Hygiene of Sex.

The importance of instructing children and young people in the psychology and hygiene of sex is practically agreed to by most teachers and authorities. But the specific matter and method of instruction, and the practicability of introducing sex instruction in our schools and colleges is not so well determined upon.

As an introduction to the series of talks on the specific instruction to be taught the child, that will appear each month in this department, we are fortunate to be able to present

THE REPORT OF THE COMMITTEE OF THE AMERICAN FEDERATION FOR SEX HYGIENE, which represents the expression of our best authorities on this subject.

"In general harmony with the propositions referred to us, and in the light of this body of expert opinion and criticism, your committee ventures to offer the following general outline of a plan of sex education:

"1. Sex instruction has a purely practical aim, and should be strictly limited by this aim. Its purpose is to impart such knowledge of sex at each period of the child's life as may be necessary to preserve health, develop right thinking and control conduct. Its aim is both hygienic and ethical, and whatever knowledge of sex and of sex relations in human life is not necessary at any particular period of the child's life for these ends should not be imparted at that period. In all cases, however, temptations should be anticipated by the instruction necessary to protect the child from physical or moral harm.

"A further aim of such instruction, not only to children, but also to adults, is to develop a healthier public sentiment in regard to sex, which will make it possible to discuss with more freedom than is now customary the grave hygienic and moral dangers to the individual and the community which grow out of the violation of the physical and moral laws governing sex life and the sacred processes of human reproduction. The common reluctance of educated people in the past to tolerate discussion of this subject outside of medical circles must give way, and is rapidly giving way, to a nobler and purer sentiment which will recognize that whatever is fundamental and vital to health and morals in the individual and the community is a proper subject for serious thought and discreet discussion. Moreover, a knowledge of the marvelous processes by which life is perpetuated, from its lowest forms to its highest, impresses on the mind more firmly than is possible in any other way the sacredness of human reproduction and the dire consequences to future generations of wrong sexual conduct.

"2. Thus limited by its aim, sex instruction must differ in one important respect from other scientific instruction, in that it must not seek to create interest and awaken curiosity in the subject with which it deals, but merely to satisfy the curiosity which spontaneously arises in the child's mind, by answering his questions truthfully but only so completely as may be necessary to give proper guidance to his conduct, both hygienic and ethical. The less children and youth think of sex, and the later they mature sexually, the better, both physiologically and ethically. Premature development of the sex consciousness and sex feelings is harmful.

"Sex instruction, therefore, should aim to keep sex consciousness and sex emotions at the minimum, and should avoid everything which tends to intensify either. Natural curiosity abates when judiciously gratified in a way not to suggest further inquiry. Children, like adults, are curious, not about what they do know, but about what they do not know. In the later years of adolescence, however, such instruction may properly suggest the vast hygienic and ethical implications in the facts of sex and of reproduction. It is important also to satisfy all normal sex curiosity before it becomes reinforced by the development, at adolescence, of the sex emotions, and to anticipate the possibility of the child's receiving sex information from impure sources.

"3. It follows from the above principles that detailed descriptions of external human anatomy are to be avoided, and that descriptions of internal anatomy should be limited to what is necessary to make clear and to impress the hygienic bearing of the facts to be taught. The details of human embryology which have no direct bearing on important practical truths should likewise be avoided. In printed books and leaflets, cuts illustrating human anatomy should be avoided wherever possible, and, if used at all, should be limited to the absolutely essential facts and be conventionalized for the purpose as much as scientific accuracy will permit.

"It is both important and timely, in the judgment of your committee, that the federation should emphasize the necessity of wisdom and good judgment in imparting sex instruction and in the publication of booklets and leaflets on the subject. Danger to the movement might conceivably come from lack of judgment or excess of zeal on the part of its friends.

"4. The purely scientific basis for such instruction must be laid in the biological nature study in elementary schools and in the more systematic instruction in biology and hygiene in secondary schools and colleges.

"5. It must be supplemented by providing proper physical exercise, by insisting in the home on regular hours of sleep, by providing adequate facilities for play and wholesome amusements, by protecting children from the unwholesome associations and corrupting influences of debasing shows and immoral literature, and by maintaining the confidence of children in their parents and teachers, so that signs of danger may be the more promptly detected.

"6. The purely scientific instruction must be reinforced as strongly as possible by ethical instruction, both direct and indirect, with due regard to the maturity of those taught. Such instruction should be incorporated in the general course in practical ethics in secondary schools and colleges, and certain phases of it should be made a part of the general lessons in morals in the upper classes of the elementary schools. Appeals to the sense of personal self-respect and purity, and to the instinct of chivalry, can be effectively made in the earliest years of adolescence, and even before. With relatively mature students, the vast sociological bearings of the subject, with their ethical implications, can be effectively utilized.

"Among the means of indirect ethical instruction for this purpose, the most effective is good literature. It is of immense consequence that during the adolescent years the pupils' minds be saturated with the great masterpieces, both in poetry and prose, which deal with romantic love in its purest forms. Thought of sex and sex emotion must at this time be spiritualized and placed on the highest plane, and good literature is the most effective means to this end which is available in the public schools. Any kind of sex education which ignores the education of the emotions is seriously defective. Deep intellectual interests, enthusiasm in art, or ardent devotion to some worthy practical cause, absorb the mind and furnish wholesome avenues for the expression of the emotions. Few conditions are so dangerous at this period as idleness, whether physical or mental, and an absence of interest in things which appeal to the higher altruistic instincts.

"7. The value of physical exercise, especially in the form of play and athletic sport, in its bearing on the control of the sex instinct, is so generally recognized that it needs no special emphasis here.

"8. For the purpose of outlining more specifically the character of the instruction adapted to various ages, the life of the pupil may be divided conveniently into four periods, namely, from one to six, from six to twelve, from twelve to sixteen, and from sixteen to full maturity. This division, as will be recognized, is not wholly arbitrary, but rests on a basis of facts, both physiological and psychological.

"The period from one to six is the period preceding admission to school, and is therefore the only period during which the care of the child falls chiefly upon the mother—the kindergarten at present reaching only a small proportion of children. It is therefore important that in lectures on sex education given to mothers special emphasis be laid upon this period, and that proper instruction be given as to the care of the child's body. The danger to the child of placing it in the care of an immature or injudicious nurse should be pointed out. Instruction should be given as to how the child's questions relating to the origin of human life may best be answered. This is the only sex instruction a child needs during this first period. Watchfulness over the child's habits, and protection from untoward influences, constitute the mother's chief duty.

"10. The period from six to twelve, which might be subdivided into that of early childhood and that of later childhood, covers the greater part of the elementary school period. Here the school must share with the home the hygienic and moral care of the child; and, as most parents are not qualified at present to give the necessary sex instruction to their children, this duty falls mainly upon the school.

"Truthful and delicate answering of the child's questions as to the origin of the individual human life, and instruction which will protect it from forming injurious sexual habits, constitute the chief features of sex instruction during the early years of this period. Such instruction at this period is best given privately, and should be carefully adapted to the child's individual needs.

"It is essential at this, as at other periods, that the child be provided with abundant facilities for play and exercise; that his habits of sleep be regular, and that he be protected from corrupting social influences at school and in the neighborhood.

"In addition to the above, there shall be given, during the years of later childhood, including the remaining years of the ordinary elementary school course, a carefully planned series of lessons on reproduction in plants as a part of the course in nature study. The child should be made to understand the function of root, leaf, flower and seed; the different modes of scattering seeds; the various methods of fertilization and the necessity of fertilization; and he should be led up to the generalization that plant life always springs from plant life.

"In like manner, a series of lessons on reproduction in animal life below mammals should be given, making use of familiar animals. The origin of the chick, the fish and the frog from the egg, and the metamorphosis of the frog; the origin of insects and their metamorphoses; and, finally, the necessity for fertilization—these might form the chief general topics of such a series of lessons.

"The aim should be, so far as specific sex instruction is concerned, to impress deeply the mind of the child with the beautiful and marvelous processes of nature by which life is reproduced from life, both in the plant world and in the animal world. It is not necessary, and in most cases not desirable, that children should make application of this knowledge to reproduction in man before the beginning of adolescence, further than that the human infant is developed within the mother. But such instruction on reproduction in nature will create the background of knowledge which will afterward invest reproduction in the higher animals and in man with a significance and a dignity not otherwise attainable, and, what is equally important, it will create the right emotional attitude toward human reproduction and prepare the child's mind to appreciate its sacredness.

"During the last three years of the elementary school course, over-age children and children who have contracted injurious sex habits often, in addition to the above, need instruction of a special kind. Such instruction should be given privately.

"11. During the early adolescent period, approximately from the age of twelve to sixteen, reproduction in plants and in animals below the mammal should be more extensively studied, and the wonderful variety of modes of fertilization, especially in plants, be emphasized. It is important to make the pupil acquainted with a wide range of facts in order to impress his mind with the wondrous beauty of nature's provision for the perpetuation of life, the aim being always ethical as well as scientific and hygienic.

"With this background of knowledge, reproduction in mammals may be taken up. The teaching ought now to impress, with many illustrative facts, the generalization that animal life comes from *the ovum.* (The more accurate formulation may be left until later.) Fertilization in mammals should now be taught, and this should by natural steps lead up to reproduction in man. The simplest facts in regard to heredity should now be taught, and their applications be made to human life. The pupil will then be in a position to understand the significance of sexual morality, and to be impressed with the dangers to health and morals of abnormal sexual habits. Specific instruction in regard to sexual morality will now be especially effective.

"As girls mature from a year to a year and a half earlier than boys, they should receive such instruction somewhat earlier, and emphasis should be laid upon instruction in regard to the special care of their health at the change of life called puberty.

"12. In connection with the study of reproduction, both during the earlier and the later adolescent period, emphasis should be laid on the broader ethical implications of biological facts as revealed in evolution. The evolution of care for the young, of the corresponding psychic factor of love of offspring, should be made clear and be emphasized. The evolution of parental love should be traced, both in animals and in man; the facts that as the offspring become fewer and are more helpless at birth, parental love and care must of necessity increase; that the pairing of animals for this purpose foreshadows the human family; that as the human infant is one of the most helpless animals at birth and has a long period of maturing, human parental love is naturally strongest, especially in the mother. At this point biology and ethics are so closely interrelated that they can be made mutually to reinforce one another, and the significance of the family and the sacredness of the home can be impressed as in no other way that is available in the public schools.

"The ethical relations in the home between parents and children and between brothers and sisters should be emphasized. It should be impressed upon every boy that every girl is somebody's daughter and usually somebody's sister, and that it is his sacred duty to accord her the same respect and protection which he would exact from another boy toward his own sister. It has been found by actual experience that this point of view can be made to appeal strongly to boys even when some other points of view do not appeal effectively.

"13. During the entire period of adolescence, children should be given a general knowledge, not only of the physical changes through which they are passing and their hygienic and ethical significance, but they should also be led to comprehend the significant psychic, especially the emotional, changes through which they are passing. At this period children are at times a mystery to themselves, and are apt to feel that they are not understood, or are misunderstood, by their elders. If at this period parents do not maintain close sympathetic and confidential relations with them, there often result alienation of the children and loss of control by the parents.

"14. During the adolescent period, in addition to the indirect moral training through literature, as suggested above, there should be systematic instruction in practical ethics, of which the ethics of the sex relation should form a natural and integral part.

"15. During the later period of adolescence—that is, from the age of about sixteen to complete maturity, there should be given, in addition to the foregoing, more thorough instruction in heredity and the bearing of sexual morality and immorality on future generations, and special instruction as to the character and the dangers of venereal diseases. While in individual cases such instruction is sometimes needed earlier and should be given privately, the general presentation of the subject should be reserved until later adolescence, on the general principle that any particular phase of sex instruction should be given only when it is needed as a protection from real harm.

"16. It is not desirable that there should be specially set lessons in sex instruction in schools, but such instruction, in all but exceptional cases, should form a natural part of the courses in nature study, biology, hygiene and ethics.

"17. All instruction in reproduction in plants and in animals below mammals can be given in coeducational classes. Instruction in reproduction in mammals may best be given in separate classes, and instruction in human reproduction should always be given in separate classes, and each class should be taught by a teacher of their own sex.

"18. If certain pupils need instruction beyond the needs of the class as a whole, such instruction should in all cases be given privately. The extent to which general instruction in human reproduction and sexual morality may with advantage be given in class instead of privately depends so much on the character and maturity of the class and on the personality of the teacher that no general rule can be laid down. It must be left largely to the tact and judgment of the teacher.

"19. While sex instruction in human life should not be attempted by all teachers, but should be given by those who have special qualifications for it, both personal and academic, it is not desirable that any teacher should make a specialty of this type of instruction and of no other. We do not want "sex specialists" any more than we want special courses in sex instruction detached from the courses in science and ethics of which they naturally form an integral part. All that should be demanded of such a teacher is that he should have a thorough and accurate knowledge of the scientific facts involved, and possess the personal qualities and the teaching tact and skill necessary for effectively presenting the subject. Other things being equal, it is better that one of the regular corps of teachers in the elementary schools, whom the pupils personally know and who has a firm moral hold on them, should give this instruction, than that a specialist who is a stranger should come in and be known to the pupils as the teacher of this subject alone. In secondary schools and in colleges, such instruction would naturally be given by the teachers of biology, of hygiene and of ethics.

"20. In order that some teachers possessing the necessary personal qualifications may be trained to give this instruction, and in order that all teachers may have such general knowledge of it as to be in intelligent sympathy with special teachers and lend them their support, all normal schools and all departments of education in colleges and universities should provide the necessary courses to meet this need.

"21. As many pupils do not enter a secondary school, or even complete the course in the elementary schools, it is essential that sex instruction be given in the regular evening schools which such pupils now generally attend in most cities of the country. Such instruction in evening schools, because of the shortness of the term of such schools, may profitably emphasize the human and most vital aspects of the subject somewhat at the expense of its broader biological treatment. It is essential that such instruction cover the topics of most vital importance to the pupil, and not stop short of that by devoting too much of the limited time to more or less preliminary topics. It is needless to add that for purposes of this instruction students must be classified according to age and not according to general scholarship.

"22. As parents should naturally give much of this instruction to their own children, and as comparatively few of them are qualified to give it, systematic courses of lectures should be provided for them at public expense.

"23. Courses in sex instruction, carefully adapted to the age of those taught, should be organized in Young Men's Christian Associations and in various types of boys' clubs. Such classes have, in fact, been formed in many such associations and clubs.

"In conclusion, your committee would emphasize the necessity of good judgment and tact in introducing sex instruction into schools. It should not be introduced prematurely, but only so fast as teachers can be found or trained who are competent to give it, and so fast as public sentiment will support it. On the other hand, undue weight must not be given to the difficulties attending such instruction even under present conditions, inasmuch as even occasional mistakes will do far less harm than allowing children to continue to gain this knowledge, as many of them now do, from impure sources—a pernicious first impression which induces in them an attitude of mind toward the subject that makes it extremely difficult to give them later the best instruction. In not a few such cases subsequent sound teaching is practically fruitless.

Respectfully submitted,

THOMAS M. BALLIET,
MAURICE A. BIGELOW,
PRINCE A. MORROW,
Committee."

